# The unbearable uncertainty of panarthropod relationships

**DOI:** 10.1098/rsbl.2022.0497

**Published:** 2023-01-11

**Authors:** Ruolin Wu, Davide Pisani, Philip C. J. Donoghue

**Affiliations:** ^1^ Bristol Palaeobiology Group, University of Bristol, Tyndall Avenue, Bristol BS8 1TQ, UK; ^2^ School of Earth Sciences, University of Bristol, Tyndall Avenue, Bristol BS8 1TQ, UK; ^3^ School of Biological Sciences, Life Sciences Building, University of Bristol, Tyndall Avenue, Bristol BS8 1TQ, UK

**Keywords:** Panarthropoda, Tactopoda, Lobopodia, Protarthropoda, phylogeny, topology testing

## Abstract

Panarthropoda, the clade comprising the phyla Onychophora, Tardigrada and Euarthropoda, encompasses the largest majority of animal biodiversity. The relationships among the phyla are contested and resolution is key to understanding the evolutionary assembly of panarthropod bodyplans. Molecular phylogenetic analyses generally support monophyly of Onychophora and Euarthropoda to the exclusion of Tardigrada (Lobopodia hypothesis), which is also supported by some analyses of morphological data. However, analyses of morphological data have also been interpreted to support monophyly of Tardigrada and Euarthropoda to the exclusion of Onychophora (Tactopoda hypothesis). Support has also been found for a clade of Onychophora and Tardigrada that excludes Euarthropoda (Protarthropoda hypothesis). Here we show, using a diversity of phylogenetic inference methods, that morphological datasets cannot discriminate statistically between the Lobopodia, Tactopoda and Protarthropoda hypotheses. Since the relationships among the living clades of panarthropod phyla cannot be discriminated based on morphological data, we call into question the accuracy of morphology-based phylogenies of Panarthropoda that include fossil species and the evolutionary hypotheses based upon them.

## 1. Introduction

Euarthropods (Chelicerata, Myriapoda and Pancrustacea—also referred to as Arthropoda [1]) dominate animal biodiversity but the origin of their bodyplans remains unclear due to the uncertainty of phylogenetic relationships with their nearest living relatives. Euarthropods are members of Ecdysozoa, a clade composed of Scalidophora (Kinorhyncha, Lorcifera and Priapulida), Nematoida (Nematoda and Nematomorpha) and Panarthropoda (Euarthropoda, Onychophora and Tardigrada). Conventionally, molecular [2–6] and some morphological [7–13] phylogenetic analyses have supported the Lobopodia hypothesis (=Arthropoda of [1]) in which Euarthropoda and Onychophora are closest relatives; however, this has been challenged by morphology-based phylogenetic analyses that instead support a sister-group relationship between Euarthropoda and Tardigrada (Tactopoda hypothesis) [10,14–20]. The Protarthropoda hypothesis (a clade of onychophorans and tardigrades) is a third rival that has been supported by both molecular [21,22] and morphological [19,23] data. These competing hypotheses impact upon attempts to resolve the relationships of fossil and living ecdysozoans and, consequently, result in contrasting scenarios for the evolutionary assembly of panarthropod bodyplans.

Since support for Tactopoda is rooted in morphology and attempts to resolve bodyplan evolution require integrated phylogenetic analysis of living and fossil taxa, here we explore support for these competing phylogenetic hypotheses within morphological datasets that have recovered Lobopodia [8,9,13] and Tactopoda [16–18]. Morphology-based phylogenetic analyses are particularly sensitive to taxon and character sampling, as well as methods of phylogenetic inference, principally because of their small size. Through application of parsimony, maximum likelihood and Bayesian phylogenetic inference methods as well as standard statistical tests of phylogenetic support, we show that morphological datasets cannot discriminate among the three competing phylogenetic hypotheses of panarthropod relationships. As such, we should anticipate that the relationships of their fossil relatives and the evolutionary narratives based upon them, are even more weakly supported.

## 2. Material and methods

To determine whether morphological datasets can discriminate among the three competing phylogenetic hypotheses, we analysed morphological datasets that have been used previously to support them [9,13,18] using parsimony, maximum likelihood and Bayesian methods of phylogenetic inference. Within these frameworks, we explored the sensitivity of the optimal topologies to the data on which the hypotheses are based. We did this by constraining the phylogenetic analyses to the Tactopoda, Lobopodia and Protarthropoda hypotheses.

We evaluated statistical support for these competing topologies using Kishino-Hasegawa (KH) [24], Shimodaira-Hasegawa (SH) [25], approximately unbiased (AU) tests [26] and Bayes factors (BFs) implemented using the stepping stone method [27] or harmonic mean [28]. These tests determine whether, given the data and the model, phylogenetic hypotheses can be distinguished from one another. This approach is particularly important in morphological and palaeontological datasets because of their comparatively small size relative to molecular datasets and the expectation that decisiveness correlates with dataset size [29].

### (a) Datasets

A diversity of morphological datasets have been used to resolve panarthropod relationships, but most of these are members of three dataset families, two supporting Lobopodia, the other supporting Tactopoda. As exemplar Lobopodia-supporting datasets, we used Legg *et al*. [9] (henceforth 'Legg dataset'), updated from Legg *et al*. [8] and Rota-Stabelli *et al*. [22], and Aria *et al*. [9] (henceforth 'Aria dataset'), modified from Aria [25]. The Legg dataset is composed of 311 taxa and 753 characters, including 90 extant euarthropods, two extant onychophorans and two extant tardigrades, plus *Caenorhabditis* and *Priapulus* as outgroup taxa. The Aria dataset is composed of 111 taxa and 276 characters, including 36 extant euarthropods, plus Nematoda and Priapulida as the outgroup; the clades of onychophorans and tardigrades are distinguished as 'Onychophora' and 'Tardigrada'. As an exemplar Tactopoda-supporting dataset, we used Yang *et al*. [18], updated from Yang *et al*. [17] and Smith & Ortega-Hernandez [16] (henceforth 'Yang dataset'). The Yang dataset is composed of 50 taxa and 95 characters, including two extant euarthropods, three extant onychophorans and five extant tardigrades, plus *Tubiluchus troglodytes* as an outgroup.

### (b) Phylogenetic methods

To control for the impact of competing phylogenetic inference methods, we used PAUP* 4.0 [30] to perform parsimony analyses; Iqtree 2.1.3 [31] to perform maximum-likelihood analyses; and MrBayes v. 3.2.7a [32] to perform Bayesian analyses. For parsimony, characters are unordered and equally weighted. For maximum likelihood, we used the Mk + FQ + R3 model for the Legg dataset, the Mk + FQ model for the Yang dataset and the Mk + FQ + ASC + G4 model for the Aria dataset. For Bayesian, we used the Mkv+Г model (Mk and Mkv model see [33]). These maximum likelihood models are the best-fitting models identified for each dataset by Modelfinder [34] in lqtree according to Bayesian information criterion (for analytical detail see the electronic supplementary material).

### (c) Topology tests and model selection method

**Figure 1.**
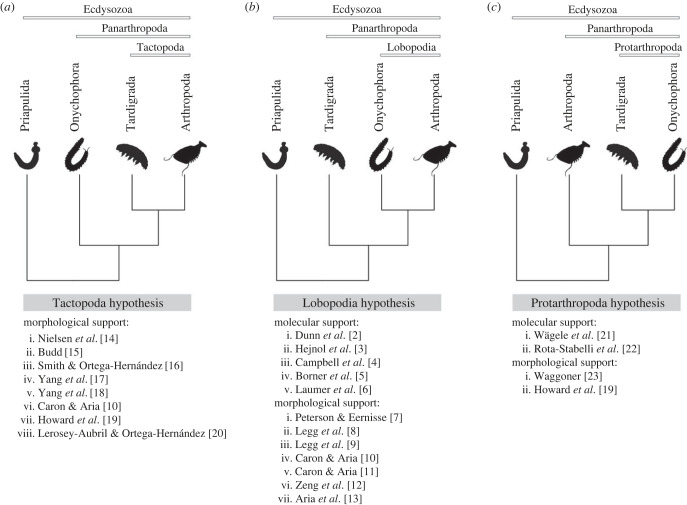
Competing hypotheses within panarthropod lineages and a summary of the support for the topologies of the hypotheses. (*a*) Tactopoda hypothesis, (*b*) Lobopodia hypothesis, (*c*) Protarthropoda hypothesis. Major related studies are listed and details see corresponding references.

In an attempt to discriminate among the competing hypotheses, we first conducted unconstrained phylogenetic analyses of the morphological datasets using each of the phylogenetic inference methods. We then undertook three constrained analyses in which partial (backbone) topology constraints were imposed upon the relationships of the living species only; positions of fossil species were unconstrained in all instances. These topological constraints were implemented to be compatible with the three competing hypotheses of panarthropod relationships (figure 1).

To determine whether the data can discriminate decisively between hypotheses, we investigated support levels across the considered topologies and implemented tests to compare pairs of alternative tree topologies. Node support was estimated using bootstrap (1000 replicates) under parsimony and maximum likelihood. For the Bayesian analyses, node support was estimated using posterior probabilities.

To test alternative tree topologies under parsimony and maximum likelihood, we used the KH, SH and AU tests, using *p* = 0.05 as a significance threshold. For the Bayesian analyses, BFs were used to test the relative fit of alternative tree topologies to the data (given the model). For Yang and Aria datasets, we ran stepping-stone analyses [27] to obtain the marginal likelihood values and calculated BFs from their ratio. This approach could not be applied to the larger Legg dataset because of computational tractability and so for this dataset we estimated BFs from harmonic means [28]. BFs were interpreted following Kass & Raftery [35].

## 3. Results

### (a) Unconstrained phylogenetic analyses

Unconstrained parsimony analysis of the three datasets recovered the results reported in the papers from which the datasets were derived, Lobopodia [9,13] and Tactopoda [18] respectively. Maximum-likelihood and Bayesian analyses recovered compatible topologies, though the consensus trees from the Bayesian analyses were often less well resolved (as expected [36,37]) than the maximum-likelihood trees (see electronic supplementary material).

**Figure 2.**
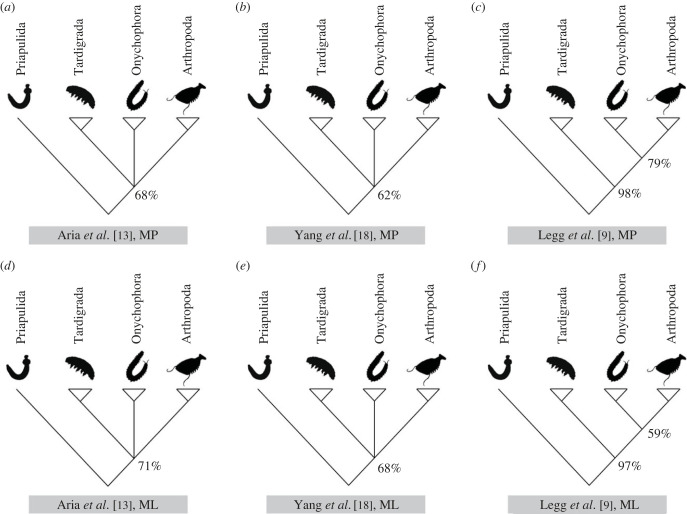
Bootstrap results of the Aria, Yang, and Legg dataset under parsimony and maximum-likelihood criterion. Numbers adjacent to nodes reflect clade support bootstrap results under the parsimony criterion for (*a*) Aria (*c*) Yang and (*e*) Legg dataset; under the parsimony criterion the bootstrap results for (*b*) Aria (*d*) Yang and (*f*) Legg dataset.

Bootstrap analyses of the two morphological datasets under parsimony and maximum likelihood generated highly unresolved consensus trees, as expected given the Bayesian analysis results (figure 2). In particular, bootstrap support values for the Yang dataset suggest that it is not possible to discriminate the relationships among the three panarthropod phyla. Bootstrap analysis of the Legg dataset recovers Lobopodia (BS-MP = 79%, BS-ML = 59%; BS: bootstrap support. Figure 2*e,f*), but the internal relationships are poorly resolved. The bootstrap consensus from the Aria dataset is highly unresolved.

### (b) Constrained phylogenetic analyses and topology tests

Under parsimony, constrained analyses of the Yang dataset yielded 4978 most parsimonious trees (MPTs) when Tactopoda was enforced, 6434 when Lobopodia was enforced, and 4034 when Protarthropoda was enforced. The KH test cannot discriminate among hypotheses. Using the KH test, under the Tactopoda constraint, all hypotheses have *p* = 1. Under Lobopodia, *p-*values range 0.3199–0.6971. Under Protarthropoda, *p-*values range 0.3199–0.6721 (table 1*f*). Under maximum likelihood, KH, SH and AU tests yielded *p-*values ranging 0.145–1.000 under all constraints (table 1*g*). Stepping stone analysis yielded BFs close to 1 for all, with a difference of less than 0.01, indicating no significant statistical difference between the competing topologies (table 1*h* and electronic supplementary material).

**Table 1.** Results of constrained analyses and topology tests by using the Aria, Yang and Legg dataset. (*a*–*d*) Results for Aria dataset, (*e*–*h*) results for Yang dataset, (*i*–*l*) results for Legg dataset. (*a*, *e*, *i*) The number of the extant species of different clades in the dataset of three datasets, (*b*, *f*, *j*) KH test result under parsimony trees of three various hypotheses, (*c*, *g*, *k*) KH, SH and AU test results under maximum likelihood criterion of hypotheses, and (*d*, *h*, *l*) the marginal-likelihood values and Bayes factors calculated from them; ‘/’ means division, T, L, P: Tactopoda, Lobopodia, Protarthropoda hypothesis.

**analyses using dataset from Aria** ***et al*.** [**13**]							
(*a*) *information of the dataset*				(*b*) *tests under parsimony*			
classification	number of extant species			hypothesis	number of parsimony trees	range of the *p*-value of Kishino-Hasegawa (KH) test	
outgroup	2			Tactopoda	1	(shortest length tree)	
Onychophora	1			Lobopodia	3	all are 1.0000	
Tardigrada	1			Protarthropoda	2	0.8353, 0.8478	
Arthropoda	36						
(*c*) *tests under maximum likelihood*				(*d*) *tests under Bayesian*			
hypothesis	*p*-value			hypothesis	marginal likelihood	Bayes factor	
	KH test	SH test	AU test			ratio of models	value
Tactopoda	0	5.00 × 10^−5^	3.07 × 10^−6^	Tactopoda	−4547.380	T/L	≈1.001
Lobopodia	1	1	1	Lobopodia	−4543.720	L/P	≈0.999
Protarthropoda	0	0	2.23 × 10^−8^	Protarthropoda	−4547.410	T/P	≈1.000
**analyses using dataset from Yang *et al*.** [**18**]							
(*e*) *information of the dataset*				(*f*) *tests under parsimony*			
classification	number of extant species			hypothesis	number of parsimony trees	range of the *p*-value of Kishino-Hasegawa (KH) test	
outgroup	1			Tactopoda	4978	All are 1.0000	
Onychophora	3			Lobopodia	6434	0.3199–0.6971	
Tardigrada	5			Protarthropoda	4034	0.3199–0.6721	
Arthropoda	2						
(*g*) *tests under maximum likelihood*				(*h*) *tests under Bayesian*			
hypothesis	*p*-value			hypothesis		Bayes factor	
	KH test	SH test	AU test		marginal likelihood	ratio of models	value
Tactopoda	0.743	1.000	0.856	Tactopoda	−708.740	T/L	≈0.997
Lobopodia	0.257	0.257	0.145	Lobopodia	−710.730	L/P	≈1.002
Protarthropoda	0.257	0.257	0.145	Protarthropoda	−709.240	T/P	≈0.999
**analyses using dataset from Legg *et al*.** [**9**]							
(*i*) *information of the dataset*				(*j*) *tests under parsimony*			
classification	number of extant species			hypothesis	number of parsimony trees	range of the *p*-value of Kishino-Hasegawa (KH) test	
outgroup	2			Tactopoda	4	0.4114–0.5741	
Onychophora	2			Lobopodia	9	all are 1.0000	
Tardigrada	2			Protarthropoda	12	0.2280–0.4331	
Arthropoda	90						
(*k*) *tests under maximum likelihood*				(*l*) *tests under Bayesian*			
hypothesis	*p*-value			hypothesis	harmonic mean	Bayes factor	
	KH test	SH test	AU test			ratio of models	value
Tactopoda	0.346	0.553	0.380	Tactopoda	−9441.53	T/L	≈1.001
Lobopodia	0.378	0.488	0.403	Lobopodia	−9432.82	L/P	≈1.000
Protarthropoda	0.654	1.000	0.637	Protarthropoda	−9435.85	T/P	≈1.001

Constrained analyses of the Legg dataset using parsimony yielded four MPTs when enforcing Tactopoda, nine when enforcing Lobopodia, and 12 when enforcing Protarthropoda. Trees obtained under Lobopodia are shorter. Again, KH tests could not discriminate among hypotheses. *p*-Values for the Tactopoda trees vary 0.4114–0.5741, while the Protarthropoda trees range 0.2280–0.4331 (table 1*j*). KH, SH and AU tests could not discriminate among the Tactopoda-, Lobopodia- or Protarthropoda-constrained maximum-likelihood trees, yielding *p-*values ranging 0.346–1.000. *p*-Values for Protarthropoda are always the highest for each of the topology tests (table 1*k*). BFs from constrained analyses of the Legg dataset are all close to 1, indicating no significant difference between the competing topologies (table 1*l* and electronic supplementary material).

Constrained parsimony analyses of the Aria dataset yielded one MPT when enforcing Tactopoda, three for Lobopodia and two for Protarthropoda. Under maximum parsimony, KH tests cannot discriminate among hypotheses, while all MPTs for Lobopodia have *p* = 1 and two MPTs for Protarthropoda are 0.8353 and 0.8478 (table 1*b*). However, under maximum likelihood, KH, SH and AU tests discriminate the Lobopodia (*p* = 1) from the other (*p <* 0.05, table 1*c*). BFs for the three hypotheses are close to 1, meaning that no significant difference could be identified between any two of them (table 1*d* and electronic supplementary material).

## 4. Discussion

Resolving evolutionary relationships among the panarthropod phyla is integral to understanding the evolutionary assembly of the arthropod bodyplans, from genomic, developmental and phenotypic perspectives. The pattern of character assembly for the arthropod bodyplans is perhaps the best resolved for all fossil groups, thanks to Cambrian fossil Konservat-Lagerstätten which are dominated by euarthropods [38–40]. This is largely a consequence of the biased preservation of their recalcitrant cuticles and because many key features of the euarthropod bodyplan are represented in their cuticular skeletons [41,42]. However, it is difficult to rationalize the evolutionary significance of such data without first resolving the phylogenetic relationships among the fossil species and this depends, in turn, upon resolution of the evolutionary relationships among their extant relatives. At present, panarthropod evolution is interpreted within three competing and mutually exclusive hypotheses, Lobopodia, Tactopoda and Protarthropoda, which have different implications for the assembly of panarthropod bodyplans [39].

We have attempted to discriminate among these competing hypotheses using morphological datasets that have supported Lobopodia and Tactopoda. While it is clearly possible to recover Tactopoda from the parsimony analyses of the Yang dataset and Lobopodia from the Legg and Aria datasets, the phylogenetic signal for these hypotheses is weak in all datasets. The consensus tree from bootstrap analysis of the Yang dataset is uninformative on the relationships of arthropods, onychophorans and tardigrades. The Legg dataset also has a poorly resolved bootstrap consensus but it retains support for Lobopodia (BS-MP = 79%, BS-ML = 59%). Bootstrap analyses of the Aria dataset reveal that it is uninformative under parsimony and maximum likelihood. Bootstrap analyses are harsh tests of the signal in morphological datasets which are usually small in comparison to molecular datasets. Consistent with results from the bootstrap analyses, the results of our topology tests, designed to determine whether a dataset can statistically discriminate between alternative hypotheses, are even less encouraging. We employed KH, SH, AU and BFs to determine whether the Yang, Legg and Aria datasets can discriminate statistically between Tactopoda, Lobopodia and Protarthopoda. Our results show that the Yang and Legg datasets cannot discriminate statistically between any of the prior hypotheses, while the Aria dataset enjoys statistically significant support for Lobopodia, but only under maximum likelihood.

If the relationships of living panarthropods cannot be resolved using morphological data, what store should be put in phylogenies of their fossil relatives, on which our understanding of the assembly of panarthropod bodyplans is based? To be sure, fossils are integral to this endeavour, revealing cryptic homologies among living relatives and, therefore, informing on their evolutionary relationships [43]. Fossils also inform on the pattern and sequence of character evolution, as well as enriching understanding of historical biogeography otherwise based solely on living species [43]. However, this counts for naught unless there is a robust phylogenetic framework for living and fossil species on which to base evolutionary inferences. Two out of the three tested datasets could not resolve panarthropod relationships under any circumstances, suggesting that, minimally, morphological datasets should routinely be tested to evaluate their decisiveness, particularly when used to propose new phylogenetic hypotheses.

Rather than present counsel despair, we highlight some effective solutions to the unbearable uncertainty of panarthropod relationships. First, researchers could explore whether statistically decisive datasets of morphological characters can be assembled; not all of the datasets we analysed were designed explicitly to address panarthropod relationships and so there may be a realistic prospect that progress is possible (indeed, there is some statistical support for Lobopodia in the Aria dataset). Second, rather than striving for fully resolved but weakly supported phylogenetic hypotheses, palaeontologists and other morphologists should embrace uncertainty, inferring evolutionary history based on those statistically robust relationships that can be resolved. Third, morphology is not the only source of pertinent data and phylogenetic analyses of living and fossil panarthropods can be conducted within the constraint of phylogenetic hypotheses informed by molecular data. Molecular phylogenetic analyses have supported a diversity of hypotheses of ecdysozoans intra-relationships, particularly in terms of the phylogenetic position Tardigrada (sister to Lobopodia [4,6] versus sister to Nematoda [2,3,5,6]). However, Lobopodia is almost universally supported by current phylogenomic datasets, irrespective of whether or not Tardigrada is recovered as a member of Panarthropoda [2–6]. Based on current evidence we suggest that Tactopoda should be considered unsupported and Lobopodia, which is supported by multiple lines of evidence, should be the preferred working hypotheses for panarthropod relationships. Finally, perhaps the most progressive and effective solution is to stop discriminating between molecular and morphological data and instead marshall all data relevant to the phylogenetic question. Methods for combined analysis of morphological and molecular data are now widely available (e.g. [31]) and can be used to provide an integrated understanding of the evolutionary relationships and evolutionary history of Panarthropoda.

## Data Availability

Our paper used three datasets from published paper: the first one is Aria *et al*. [13], the second is Yang *et al*. [18], and the last one is Legg *et al*. [9]. The data are provided in the electronic supplementary material [44].
